# Evaluating the Electroencephalographic Signal Quality of an In-Ear Wearable Device

**DOI:** 10.3390/s24123973

**Published:** 2024-06-19

**Authors:** Jeremy Pazuelo, Jose Yesith Juez, Hanane Moumane, Jan Pyrzowski, Liliana Mayor, Fredy Enrique Segura-Quijano, Mario Valderrama, Michel Le Van Quyen

**Affiliations:** 1 Laboratoire d’Imagerie Biomédicale (LIB) Inserm U1146, Sorbonne Université, UMR7371 CNRS, 15 Rue de l’Ecole de Medecine, 75006 Paris, France; jeremy.pazuelo@inserm.fr (J.P.); jy.juez@uniandes.edu.co (J.Y.J.); hanane.moumane@inserm.fr (H.M.); 2 Department of Emergency Medicine, Medical Unversity of Gdańsk, 80-214 Gdańsk, Poland; jan.pyrzowski@gumed.edu.pl; 3 ONIROS SAS–Comprehensive Sleep Care Center, Bogotá 110221, Colombia; limayorag@gmail.com; 4 Department of Electrical and Electronic Engineering, University of Los Andes, Bogotá 111711, Colombia; fsegura@uniandes.edu.co; 5 Department of Biomedical Engineering, Universidad de Los Andes, Bogotá 111711, Colombia

**Keywords:** in-ear sensors, bioelectrical signals, in-ear and scalp EEGs, wearable devices, signal quality, correlation, brain monitoring

## Abstract

Wearable in-ear electroencephalographic (EEG) devices hold significant promise for advancing brain monitoring technologies into everyday applications. However, despite the current availability of several in-ear EEG devices in the market, there remains a critical need for robust validation against established clinical-grade systems. In this study, we carried out a detailed examination of the signal performance of a mobile in-ear EEG device from Naox Technologies. Our investigation had two main goals: firstly, evaluating the hardware circuit’s reliability through simulated EEG signal experiments and, secondly, conducting a thorough comparison between the in-ear EEG device and gold-standard EEG monitoring equipment. This comparison assesses correlation coefficients with recognized physiological patterns during wakefulness and sleep, including alpha rhythms, eye artifacts, slow waves, spindles, and sleep stages. Our findings support the feasibility of using this in-ear EEG device for brain activity monitoring, particularly in scenarios requiring enhanced comfort and user-friendliness in various clinical and research settings.

## 1. Introduction

Wearable devices are increasingly present in our daily lives. Their growing development has been boosted by recent technological advances combining skin-attachable health monitoring sensors with miniaturized and high-performance recording components. One attractive type of recent wearable device is worn in or around the ear. These so-called “earables”, with their specific positioning on the human head, provide a unique platform for sensing a wide range of physiological parameters (e.g., body sounds, mouth, face, eye and head movements, heart rate, blood oxygen saturation, blood pressure, and respiration) [1]. This technology takes advantage of the anatomical characteristics of the ear that offer a convenient dock to host the required electronics needed to fit a wearable device [2]. Most importantly, they are discrete and unobtrusive, similar to the audio devices people commonly use, such as earphones, earbuds, or earplugs.

Due to its proximity to the head, the external ear offers an interesting spot to monitor the brain. In particular, several types of ear wearable devices have been developed for the recording of brain electrical activities, the so-called electroencephalogram (EEG), from electrodes placed in different locations within the external ear, mostly the ear canal [3,4]. The interest in this type of technology comes from the fact that these systems use small electrodes (size ~ 5 × 5 mm^2^) that are easy to wear, requiring considerably less installation time compared to a traditional EEG setup, which needs the placement of several electrodes on the scalp by a trained staff. Moreover, the tight fit of an earpiece inside the ear canal applies pressure on the electrodes, ensuring stable electrode positions and a partial reduction in the motion artifacts that typically obscure the signal quality in a conventional EEG. These advantages have promoted ear-EEG devices as a promising tool for continuously monitoring brain activities outside clinical or lab settings [5].

The first in-ear EEG device, pioneered by Looney et al. in 2011 [3], marked a significant milestone in this wearable technology for monitoring purposes. Subsequently, the recording technique and analysis of such EEG signals have been validated through over 90 published peer-reviewed scientific papers, covering various areas, including material selection, system design for practicality, and signal quality verification [6]. Several innovative sensor designs have been proposed and validated, such as those integrating custom 3D mold impressions to better conform to ear morphology [7] or memory foam substrates [8]. Further work by Tabar et al. [9] introduced a generic earplug made of soft silicone material, showing promising signal quality and potential for long-term EEG monitoring. The development of many of these ear-EEG systems has been driven by their application in various domains. For example, ear-EEG devices have been explored for emotion [10] and stress [11] monitoring, the estimation of hearing thresholds for audiometry assessment [12], personal authentication in security contexts [13], and drowsiness detection during driving [14], among others. In physiological and pathological contexts, ear-EEG prototypes have been investigated for sleep assessment, including comparisons with gold standard polysomnography for reliable sleep scoring [15]. Additionally, ear-EEG technology shows promise in epilepsy, where in-ear EEG systems can be integrated as routine clinical tools for continuously monitoring epileptic activities outside clinical facilities, thereby enhancing the ability to capture pathological signals for improved diagnosis and follow-up [16,17]. Moreover, recent studies have demonstrated the reliability of in-ear EEGs for the long-term monitoring of epileptic activities in patients with Alzheimer’s disease [18] and Lewy body dementia [19], who are at higher risk of developing epileptic discharges. Taken together, in-ear EEG technology holds the potential for developing new monitoring procedures for various clinical conditions.

In-ear EEG signals from the ear canal are comparable to EEG signals recorded at scalp electrodes within the ear proximity during cognitive tasks [20] or sleep [21]. Nevertheless, the recorded signal inside the ear has a lower amplitude than the scalp EEG, presumably due to the greater distance from the generating sources inside the brain to the recording sites, as well as by the electrical and geometric properties of the electrodes used for its acquisition. Recording bioelectrical signals from electrodes placed on the skin surface relies critically on the electrode–skin interface. Several ear-EEG studies have been performed with wet electrodes, in which conductive gel or hydrogel was applied between the electrodes and the skin [5]. In this context, skin preparation is needed, which includes skin abrasion to remove dead cells from the top layer of the epidermis, causing a decrease in the skin–electrode contact impedance. Nevertheless, the prolonged use of these wet electrodes often results in skin irritation or lesions, thus limiting their use. Clearly, dry-contact ear-EEG electrodes would increase the comfort and user-friendliness of these devices. Recently, several works have incorporated small, dry electrodes in the earpieces to improve usability at the cost of increased electrode–skin impedance, noise, and signal-quality degradation [22]. Various conductive materials have been proposed for the use of dry electrodes, including silver [23], gold [24], titanium [25], or even carbon nanotubes [11].

Ear-EEG devices usually cover a lesser spatial extent than a standard scalp EEG device, with most recorded activities originating from temporal regions [25]. Therefore, to compensate for the loss of spatial information, it is crucial to collect high-quality signals. The assessment of this quality often requires the simultaneous recording of the designed in-ear EEG system along with conventional gel electrodes placed on the scalp. For example, Looney et al. [3] proved the high coherence between an in-ear EEG electrode and the standard scalp T7-M1 electrode (ipsilateral mastoid reference) from the international 10–20 placement system, reflecting the shared activity between the temporal lobe and in-ear locations. It is important to note that, in these assessments, the earpieces were connected to an external clinical-grade amplifier to acquire ear-EEG signals. Unfortunately, whereas this setup enhances signal quality, it also limits the findings to the monitoring electronics typically employed in clinical or lab settings. Although promising, the reliability of these signal quality evaluations should be extended to dry electrodes and wireless recording modules integrated into the earpiece itself.

Although several wearable in-ear EEG devices have appeared on the market, there is still a need for the independent validation of these technologies against gold-standard, clinical-grade devices. In this regard, our study aims to evaluate the signal quality of a new and compact in-ear EEG device developed by Naox Technologies. This device comprises non-invasive dry electrodes coupled with a miniaturized electronic system that acquires EEG signals from a subject’s ear canals. We first assessed the reliability of the hardware circuit by a simulated signal test experiment (Section II B: Hardware in the loop test). Then, we compared the in-ear EEG device with the gold-standard EEG monitoring equipment using the correlation coefficient between the two traces in several physiological conditions during wakefulness and sleep. Specifically, following similar approaches that have chosen the linear correlation coefficient as a comparison variable between EEG signals and especially between those acquired with dry and wet EEG electrodes [3,26], this study aims to compute the correlations between the signals captured by the ear-EEG device and those recorded from scalp EEG electrodes located at the temporal derivations T8 (right temporal) and T7 (left temporal). From a general perspective, our approach emphasizes a comprehensive and meticulous assessment of the signal quality of an in-ear EEG device. It aims to ensure a systematic, transparent, and thorough evaluation prior to its potential application in real-world scenarios.

## 2. Materials and Methods

### 2.1. In-Ear Device

**Figure 1:**
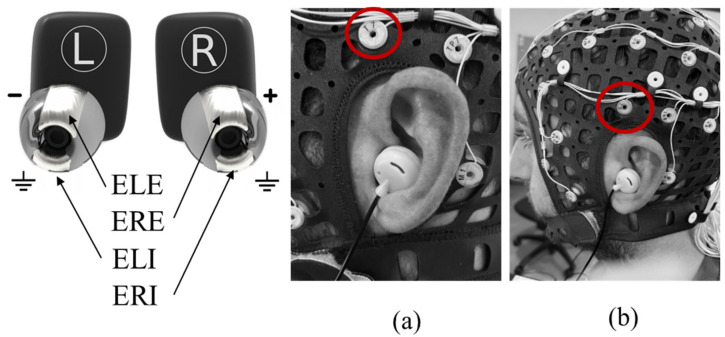
The recording setup used in this study. (**Left**) Earpieces of the in-ear device developed by Naox Technologies with electrodes inserted in the positions ELE, ERE, ELI, and ERI corresponding to two contact points inside each ear canal. (**Right**) Left-side view of the dual setup with both the scalp and the in-ear devices in place (**a**,**b**). Standard scalp T7 electrode (red circle) was used for comparison.

The in-ear EEG device was developed by Naox Technologies and followed the recommendations proposed by the scientific literature (Table 1). The electrodes for the in-ear EEG setup are hand-crafted using silicon and coated with conductive silver ink, a combination chosen for its biocompatibility and conductivity. The size of the conductive surface ranges from 8 mm^2^ to 11 mm^2^. Referring to the labels previously proposed [5], the device has four electrodes ERE, ERI, ELE, and ELI, corresponding to two contact points inside each ear canal (left and right) (see Figure 1). Following a cross-ear electrode configuration (left ear superior–right ear superior, ELE-ERE), the system operates a single bipolar EEG channel, measuring the voltage differences between the electrode located in one ear canal and the electrode in the opposite ear canal. Moreover, the GND is connected by linking the bottom electrodes of each earpiece (ELI and ERI) inside both ear canals. At 50 Hz, the electrode–skin interface has an average impedance of 459 ± 295 kΩ (*n* = 7 subjects), comparable to the impedance of a state-of-the-art in-ear dry electrode (for example, 377 kΩ in [7]). An electronic card amplifies this EEG signal and transforms it into a digital signal at a sampling frequency of 250 Hz and 24 bits resolution. A chip sends data in real time via Bluetooth low energy 2.4 GHz (BLE) to a laptop. A battery allows uninterrupted recording for 10 h, providing an extended period of continuous monitoring. Weighing around 20 g, the device provides a lightweight and unobtrusive solution for users. Moreover, the system adheres to electrical safety standards, ensuring the safety and reliability of the device in diverse usage scenarios.

### 2.2. Hardware in the Loop Test

To assess the electronic capabilities of the earbuds to correctly capture EEG signals over a wide range of amplitude and frequency bands, we opted for a real-time emulation of pre-recorded scalp EEG signals using an electronic test device designed for medical equipment (Whaleteq SEEG 100-Single Channel EEG Test Unit). This system produces arbitrary waveforms at up to ±1 V, which is then applied to a precision 1000:1 divider to produce the voltages at up to ±1 mV level (2 mVpp). After selecting an arbitrary input signal, this unit enabled us to feed it back into the earbuds’ electronic board. For these tests, we selected the Physionet Siena Scalp EEG Database [27,28,29]. This open-access dataset consists of 43 long-term EEG recordings (around 128 h) obtained from 14 subjects with epilepsy. The patients were monitored at a sampling rate of 512 Hz, with electrodes arranged based on the international 10–20 system. In total, the database contains 47 seizures, which were classified according to the criteria of the International League Against Epilepsy, and include focal onset impaired awareness, focal onset without impaired awareness, and focal to bilateral tonic–clonic seizures. Our setup focused exclusively on the T7-T8 positions, aiming to achieve the closest proximity to the intra-auricular region. We developed a script to adjust the sampling frequencies (250 Hz for in-ear data and 512 Hz for SIENA data) and precisely align the two recordings. Finally, following the seizure annotations in the database, we calculated linear correlation coefficients for 30 s windows across all ictal and interictal periods in each of the 43 recordings.

### 2.3. Scalp EEG Acquisition System

The general evaluation framework setup in this study was designed to compare the EEG signals captured by the in-ear wearable device and those recorded with the gold-standard EEG monitoring equipment with wet scalp electrodes placed at the temporal locations T7 and T8. In our work, we acquired scalp EEG signals with a 32-channel Compumedics Grael 4K-EEG amplifier at a sampling rate of 512 Hz and the Compumedics acquisition software Curry 7. We positioned scalp electrodes according to the 10–20 international system. For the resting state protocols (see *II-E*), we conducted a targeted approach, placing electrodes only at the T7 and T8 locations, along with a reference on Cz and ground electrode on the mastoid. Additionally, for the sleep protocols, we employed a comprehensive electrode configuration following the full 10–20 system. In both scenarios, skin preparation involved the application of an abrasive paste (Nuprep) and then a conductive gel (Ten20) to enhance electrode–skin conductivity. Throughout the recordings, we meticulously monitored the impedances of each electrode to ensure that they remained below 5 kΩ, thus ensuring the integrity of the recorded EEG signals. Before the experiments, we also visually inspected the EEG signal quality to discard any failure in the contact between electrodes and the skin.

### 2.4. Synchronization System

**Figure 2:**
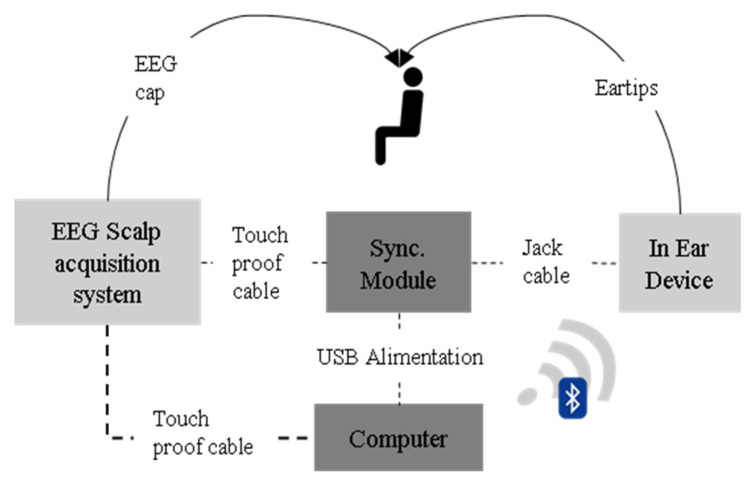
Schematic representation of the in-ear and scalp setup, with the synchronization module between the two setups. Each cable connecting the components is labeled for reference.

For our analysis, achieving a high temporal precision (below 1 ms) between the two EEG devices (scalp and in-ear) was crucial to establish correlations between the signals from both sources. To tackle this challenge, we designed an electronic board that delivered electrical pulses (50 ms duration and ~75 mV monopolar amplitude) every 10 s to both devices through dedicated auxiliary or bipolar analog inputs (Figure 2), ultimately enabling us to realign the signals effectively. The analog pulse had a duration of 50 ms and a monopolar amplitude of ~75 mV with rise and fall times set at 32 microseconds and a rise level of 30 mV. With this configuration, we were able to guarantee a precise synchronization between signals below the millisecond threshold. The recorded signals of both devices were further aligned by a software routine developed for this purpose. To achieve this, we implemented a two-step offline process: First, to detect the trigger signals, we performed a peak detection from the absolute values of the filtered signals (Butterworth lowpass filter with an order of 4 and a cutoff frequency of 20 Hz) and subsequently discretized them into a binary array. Then, this binary array served as a temporal reference, enabling the precise alignment of the recorded EEG signals from both devices.

### 2.5. Resting State Protocols

#### 2.5.1. EEG Recordings

For our experiments, we recorded 18 healthy adults with no known neurological or psychiatric disorders (Age mean ± std: 28.5 ± 5.7 years, range: 24–45 years; Male/female: 10/8) with wet scalp electrodes and the in-ear device. All participants sat comfortably in a quiet room and were about 0.5 m away from a computer screen. The experiment systematically included 7 phases (Table 2): (1) During two time periods, the participants were requested to alternatively open and close their eyes ten times for 5 min. An auditory cue indicated a change in condition every 30 s. As in standard EEG protocols, we asked the participants to stay immobile and to avoid significant head and facial muscle contractions during the recordings, thus avoiding or minimizing motion artifacts. (2) The participants were requested to perform 10× horizontal eye movements (left then right, in an alternative way) following a sound generated every 5 s. (3) Hyperventilation (HV) with a respiratory rate of approximately 20 to 30 breaths per minute, following the guidelines of the American Clinical Neurophysiology Society [30] and the British Society for Clinical Neurophysiology (ANS/BSCN Guidelines for Hyperventilation During EEG Recordings) [31]. We requested a minimum of 3 min of HV with a baseline of 2 min after HV. (4) A psychomotor vigilance task (PVT) that measures sustained attention and the consistency with which subjects respond to a visual stimulus (10 min). (5) Spoken language production testing muscle contamination of the EEG recordings (10 min). (6) A cognitive test of facial emotion recognition with motor response (5 min). (7) Relaxation period with eyes closed (10 min).

These protocols were designed to cover several resting state conditions, each exhibiting different head and facial muscle contractions levels. For quantification purposes, we organized them based on the degree of artifact contamination: First, quiet states (C1: Tasks 1 and 7) with minimal motion interference, including only eyes opening/closing. Second, active states (C2: Tasks 4 and 6) comprise two attention-demanding tests and involve intermittent subject movements (reaction to a visual stimulus and tapping on a tablet). Lastly, artifacts (C3: Tasks 2, 3, and 5) include tasks involving pronounced eye, face, or head movements. These behaviors produce large shifting voltages in both scalp and in-ear EEGs. These artifacts originated from mechanical forces applied to the in-ear electrodes, inducing small electrode movements relative to the sensors, which, in turn, cause the recorded voltage to change regardless of the cortical activity. Altogether, the experiments lasted around 60 min. All subjects provided their written informed consent before participation, and the study was approved by the ethical committee of the school of engineering of the University of Los Andes, Bogotá, Colombia.

#### 2.5.2. Data Analysis

In order to quantify the similarity of the activity in the alpha frequency band (8–12 Hz) recorded simultaneously at the scalp (contralateral bipolar T7-T8 signal) and with the Naox in-ear EEG system (180 trials of 30 s for all subjects), we evaluated the linear correlation coefficient (Pearson correlation coefficient) during the eyes-closed condition. For this, we first used a wavelet decomposition, which was computed with the continuous Gabor Wavelet [32] that is defined by the following equations:

(1) $$C(s,\tau )=\frac{1}{\sqrt{s}}\int_{-\infty }^{\infty }x(t)*\psi \left(\frac{t-\tau }{s}\right)dt$$

(2) $$\psi (t)=\frac{1}{{\left(\sigma^{2}\pi \right)}^{1/4}}exp\left(\frac{-t^{2}}{2\sigma^{2}}\right)e^{i\eta_{3}t}$$

where *t* indicates time, *s* represents scale, *τ* represents translation, $\eta_{s}$ indicates the angular frequency at scale *s*, and *σ* indicates the standard deviation of the Gaussian window in time. In our case, we defined a resolution of 0.5 Hz and *σ* = 9. It allowed us to calculate the time–frequency spectrograms of both signals for each subject. Then, from the scalp EEG, an alpha burst was detected on non-overlapping windows of 6 s if the maximum of the mean wide spectrum (3–30 Hz) (1) was found for a frequency between 8 and 12 Hz and (2) had a sufficient peak height. Based on this, we defined an alpha burst by considering a signal-to-noise ratio (SNR) above 1.5, meaning that the peak in the alpha band had to be 1.5 times higher than the rest of the spectrogram. If so, we calculated the normalized cross-correlations (“xcorr” function in MATLAB, with a lag of 0.1 s) on the filtered signal in the alpha frequency band (FIR filter). Note that the selected data were not subjected to artifact rejection, which means that both good and bad epochs were considered for the analysis.

To conduct a further characterization of the alpha bursts recorded by the in-ear device, we performed amplitude analyses on windows identified as having strong alpha activities through the steps described above. For this, we averaged the Hilbert envelopes of the filtered signals between 8 and 12 Hz over 30 s windows. We then compared the similarity of the average amplitudes and calculated the linear regression coefficient. Additionally, we compared the corresponding power spectral densities (PSDs) in the alpha frequency bands estimated by taking the squared magnitude of the fast Fourier transform.

Similarly, to investigate the patterns generated by horizontal eye movements, we calculated the linear correlation coefficient between the in-ear and scalp signals for each eye movement artifact and for each subject. Subsequently, we computed the artifact amplitude by subtracting the maximum and minimum values of each generated pattern. It is noteworthy that artifacts exceeding 250 µV for the scalp or 200 µV for the in-ear signals were excluded from further processing. This quality control criterion ensured that only artifacts within acceptable physiologic amplitude ranges were considered for the analysis.

Finally, we quantified the evolution of relative spectral power (RSP) under the three different conditions C1–C3 defined above. These relative power values of a given frequency are here defined as the ratio of the sum PSDs in this frequency to the sum PSDs in a wide frequency range (0.3 Hz, 45 Hz). This analysis was conducted for each subject using sliding windows of 10 s and for the following four frequency bands: Delta (0.5 Hz–3 Hz), Theta (3 Hz–8 Hz), Alpha (8 Hz–12 Hz), and Beta (12 Hz–35 Hz).

### 2.6. Sleep Protocol

#### 2.6.1. EEG Recordings

Using the identical equipment, as described for the Resting state protocols above, a group of 8 healthy adults (age mean ± std: 27.2 ± 3.5 years, range: 23–33 years; Male/female: 7/1) underwent full polysomnography (PSG), which included 20 EEG channels (following the 10–20 system), two electrooculograms (EOGs), two electromyograms (EMGs) on the chin, and one electrocardiogram (ECG), along with the concurrent use of the Naox earbuds. Within the recorded sessions, we included eight daytime naps and five full-night recordings for the analysis, for a total recording time exceeding 70 h. All recordings were conducted in a dedicated quiet room designed for sleep recordings. This room was authorized to be a third-party laboratory located in a Naox manufacturer facility.

Half of the recordings were manually scored in 30 s epochs by a certified sleep-scoring expert in accordance with standard criteria defined by the American Academy of Sleep Medicine (AASM) guidelines [33]. For the remaining recordings, we performed sleep stagings using the U-Sleep tool. This online platform utilizes machine learning algorithms trained on a diverse set of polysomnography data to categorize sleep stages accurately [34].

#### 2.6.2. Data Analysis

For the quantification of similarity between the scalp and in-ear EEG signals during sleep, we focused our analysis on two of the hallmark activities associated with non-rapid eye movement sleep (NREM sleep) periods, known as slow waves (SW, 0.5–4 Hz) and sleep spindles (SP, 11–16 Hz). For our analysis, we first identified windows presenting strong SW and spindle activities exclusively on scalp channels (T7-T8) and only during sleep epochs scored as NREM (N2 and N3 epochs) for consecutive, non-overlapping 6 s windows. In particular, we followed a similar methodology for the selection of windows with strong alpha activities to that described above but with adjusted threshold values. These thresholds were established following a visual inspection of the scalp signals to guarantee that the detection criteria were tailored to identify these specific EEG patterns. Subsequently, we calculated the normalized cross-correlations on the corresponding filtered signals (SW or spindle frequency bands) for all windows identified as having strong SW or spindles at scalp electrodes. For a more in-depth characterization of the SW and spindle amplitudes captured by the in-ear device, we performed additional analyses on two selected recordings with strong correlations. Here, we compared the amplitudes and power of each detected event to determine their similarity with the linear regression coefficient.

Finally, we quantified trends in the correlation between in-ear and scalp EEG signals during each sleep stage (Wake, N1, N2, N3, and REM). For this, on both devices, for every 30 s window, we computed the RSP values in 4 frequency bands (delta, theta, alpha, and beta). As a measure of concordance between the in-ear signal and the gold-standard EEG at the scalp, averaged cross-correlations were evaluated between both the RSP time series during each sleep stage. In the recorded data, the wake, N1, and N2 phases were consistently present in all recordings, averaging approximately 96 min, 16 min, and 87 min per recording, respectively. The N3 phase was observed in 11 out of 13 recordings (85%), averaging 46 min per recording. REM was found in 10 out of 13 recordings (77%), with an average duration of 49 min per recording. In total, the data indicate approximately 21 h of wake, 3.5 h of N1, 18.9 h of N2, 8.5 h of N3, and 8.2 h of REM.

### 2.7. Statistics

In order to test for the statistical significance of the linear correlation coefficients obtained between the in-ear and scalp EEG signals, we implemented a Pitman nonparametric permutation test [35,36]. Nonparametric statistical testing has been widely used in neuroimaging studies [37,38]. It has the advantages that it is not concerned with population parameters and does not depend on the knowledge of the sampled population [39]. For our analysis, we followed similar procedures as those described in the literature [38,40,41], consisting of generating surrogates by randomly shuffling short-time block intervals or trials in the time domain. In particular, we fixed one signal and randomly shuffled all corresponding 6 s intervals from the second signal. Next, we calculated the Pearson correlation coefficient between the fixed and shuffled signal intervals. As recommended [35], we repeated this procedure 1600 times (permutations) to generate a distribution of 1600 coefficient values from the corresponding permutations. We then estimated the two-sided *p*-value as the proportion of the absolute values of correlation coefficients from shuffled permutations that were larger than the true absolute value of the Pearson correlation coefficient calculated from the original data series [36]. We defined a statistical significance level for *p* < 0.05. When required, the false discovery rate (FDR) when performing multiple comparisons was controlled with the Benjamini–Hochberg method [42]. For all statistical comparisons of amplitude and power measures between reference scalp and in-ear signals, we implemented a procedure similar to that described above. In this case, we permuted all values from the original distributions of measures for the comparison, and for each permutation, we obtained the difference between the means. We then estimated the two-sided *p*-value as the proportion of the absolute values of mean differences from the permutations that were larger than the true absolute value of the mean difference calculated from the original distribution of measures. We defined a statistical significance level for *p* < 0.05. Similarly, when required, we controlled the FDR when performing multiple comparisons with the Benjamini–Hochberg method.

## 3. Results

We evaluated the electrophysiological performance of the in-ear device across various recording scenarios, and we conducted a controlled validation using a research-grade EEG system with wet electrodes in laboratory settings. This assessment first involved an emulated signal test experiment where pre-recorded known EEG signals were played back into the in-ear device. Subsequently, recordings were performed on healthy volunteers during both wakefulness and sleep periods, aiming to compare the signal quality of the in-ear system with that of scalp electrodes, with particular attention to electrodes positioned at the temporal locations T7-T8. Following this experiment design, this section presents the results of the assessments, detailing the outcomes of the comparisons across six distinct recording paradigms: (A) hardware-in-the-loop tests, (B) correlations during alpha segments, (C) correlations with eye artifacts, (D) correlations during various levels of head movements and facial muscle contractions, and (E) correlations during slow-wave and spindle events during active sleep. These correlations were interpreted based on conventional criteria: poor (<0.02), fair (0.2–0.4), moderate (0.4–0.6), substantial (0.6–0.8), and almost perfect agreement (>0.8) [43]. Statistical significance was determined using a nonparametric permutation test.

### 3.1. Hardware in the Loop Test

**Figure 3:**
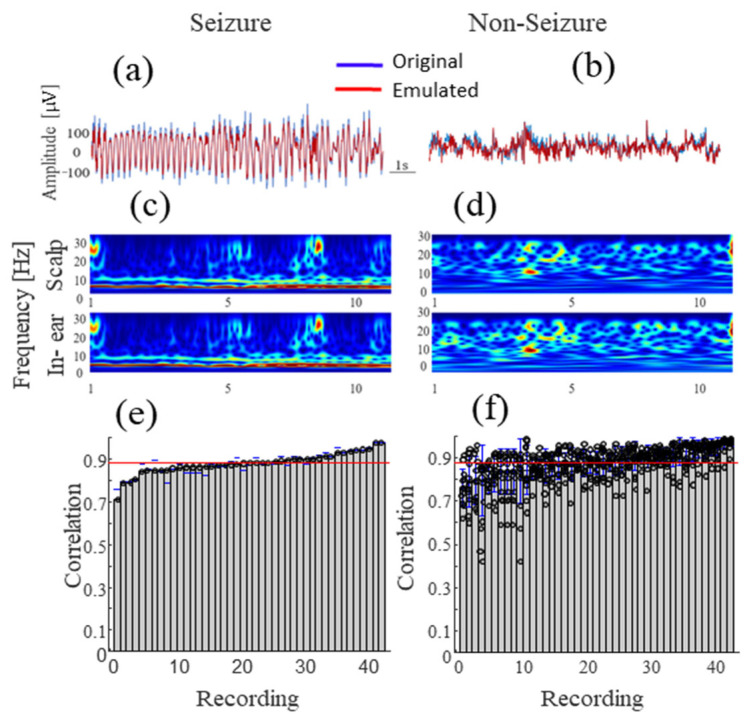
Correlation analysis between the original and emulated EEG signals during seizure (**Left**) and non-seizure states (**Right**). (**a**,**b**) A 15 s sample of original and replayed signals and (**c**,**d**) the corresponding spectrograms. (**e**,**f**) Sorted Pearson’s correlation values for each long-term EEG recording obtained from 14 subjects (45 recordings) from the Siena database.

For our study, we employed a pre-recorded EEG dataset of subjects with epilepsy comprising a total of 128 recording hours of patients replayed in real time with the Whaleteq SEEG100 system. After replaying the data, the signals collected by the in-ear device had a high degree of restoration and no apparent visual distortion, as can be appreciated in Figure 3a. In particular, Figure 3a shows a selected seizure segment in which temporal similarities between replayed and acquired signals are evident, with a perfect alignment between both. These temporal similarities are also confirmed at the spectral level, as presented in Figure 3c,d. In this figure, it is possible to observe that all time–frequency components associated with the replayed epileptic seizure are transferred to the in-ear signal. Similar observations can be drawn from the visual inspection of inter-ictal segments, as presented in Figure 3b,d. To better quantify this similarity, we then estimated the linear correlation coefficient between the replayed pre-recorded signal and acquired in-ear signal. On average, the normalized cross-correlation coefficients were 0.87 ± 0.08 ([0.71–0.99]) for window segments labeled as seizures (Figure 3e) and 0.86 ± 0.09 ([0.76–0.99]) for those labeled as non-seizures (Figure 3f). All correlations between signal segments evaluated on all 45 recordings were statistically significant (*p* < 0.05).

### 3.2. Alpha Wave Correlations

**Figure 4:**
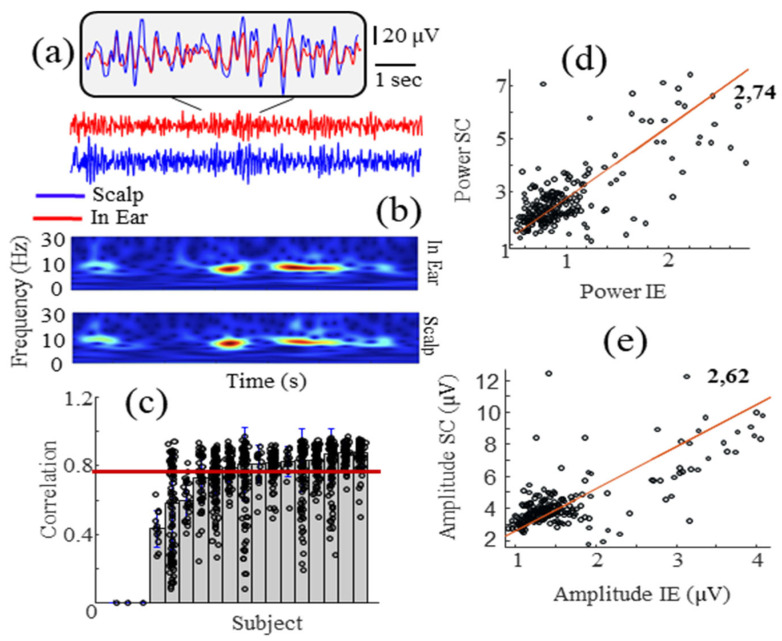
Correlation analysis of the alpha waves between in-ear and scalp EEG signals. (**a**) A 10 s sample of EEG signals during the onset of alpha waves at the scalp and the corresponding spectrograms (**b**). (**c**) Sorted Pearson’s correlation values for each subject. (**d**,**e**) Power and amplitude plots for scalp (SC) and in-ear (IE) recordings.

During this protocol, 18 participants were requested to open and close their eyes alternatively. This test was devoted to monitoring alpha waves, which are a spontaneous neural rhythms centered in the range of 7–12 Hz, especially occurring in the occipital regions when the eyes are closed and commonly used to benchmark EEG systems due to their large amplitude and prevalence in many subjects [23,25]. In this study, we evaluated the correlation coefficients between the in-ear signal and the scalp EEGs (T7-T8) to quantify the capacity of the in-ear device to record alpha waves. For the trials with a strong alpha power at the scalp (~50.4% of the 3-second-windows, 929 windows in total), we found that 87% of the subjects (13/15) had a substantial or almost perfect (>0.6) and significant (*p* < 0.05) correlation between the scalp and in-ear signals. Only two subjects had a moderate correlation. Figure 4a shows an example of a short time segment of in-ear and scalp EEG signals for a subject with a high cross-correlation (subject 8). The corresponding relative spectrogram is shown in Figure 4b with high peaks around 10 Hz, confirming the presence of the alpha wave and its high similarity between the reference scalp and in-ear signal. Across individual subjects, the results consistently demonstrate a broad range of correlations, ranging from approximately 0.43 to 0.88, with a mean value of 0.76 ± 0.19 (mean ± std) (Figure 4c). Additionally, we compared the power and amplitudes of the alpha waves recorded on both devices, evaluating their similarity with the linear regression coefficient.

**Figure 5:**
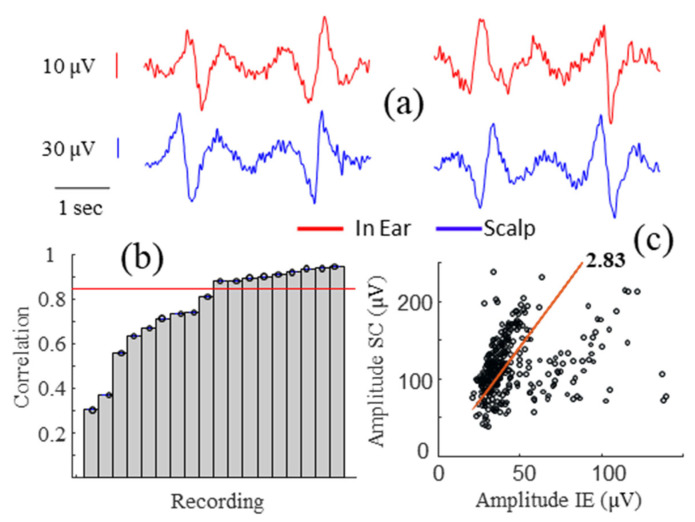
Correlation analysis of artifacts induced by horizontal eye movements. (**a**) Raw signals of artifacts (red for in-ear and blue for scalp). (**b**) Sorted Pearson’s correlation values between in-ear and scalp signals for each subject. (**c**) Amplitude plots between in-ear (IE) and scalp (SC) artifacts.

Our findings reveal that the scalp electrodes recorded a higher alpha power, averaging around 2.7 times, compared to the in-ear electrodes (Figure 4d). This observation was consistent with the created significant artifacts in temporal and in-ear EEG electrodes. Because of their stereotyped form, they provide a simple way of checking EEG system performance and stability. Figure 5a presents an example of the horizontal eye movement artifact simultaneously recorded with the scalp and in-ear systems. In this figure, the eye movement is visible in the large amplitude deviation (upward deviation followed by a downward deviation) from the background activity. For our quantitative analysis, we evaluated the correlation coefficients during artifacts induced by horizontal eye movements in 18 subjects. As illustrated in Figure 5b, we found that 83% of the subjects had a substantial or almost perfect (>0.6) and significant (*p* < 0.05) correlation between scalp and in-ear signals (mean ± std of 0.87 ± 0.19). In addition, we compared the amplitudes of the artifacts recorded on both devices, evaluating their similarity with the linear regression coefficient. We found that the scalp electrodes recorded higher artifact amplitudes, with an average that is 2.8 times greater than the in-ear electrodes (Figure 5c).

### 3.3. Correlations during Different Levels of Head Movements and Facial Contractions

**Figure 6:**
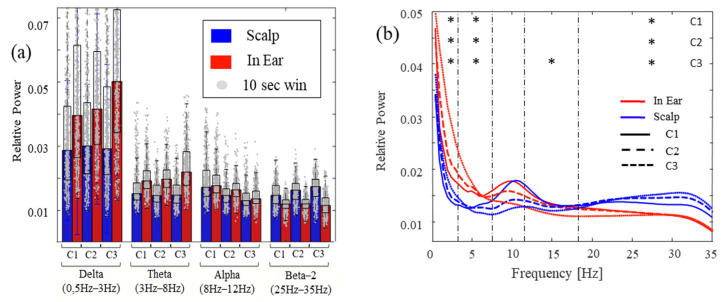
Histograms of the relative spectral powers across the four main frequency bands’ spectra (**a**) and grand-average power spectra (**b**) for in-ear (red) and scalp (blue) signals recorded during different levels of movements and facial artifacts (C1–C3). Note that, in A, outliers were removed for more clarity (* indicates a p-value of less than 0.05 for the corresponding condition C1, C2 or C3).

This protocol was developed to cover various behavioral conditions, each characterized by different levels of head movements and facial artifacts. Organized from the most stable to the noisiest, we categorized them as: C1, where participants were instructed to remain immobile and avoid facial movements. Subsequently, C2 included two concentration-demanding tasks involving some intermittent body movements. Finally, C3 involved tasks that included strong head and facial muscle contractions during the recordings. In each of the conditions (C1–C3), we explored the similarities and differences between the two devices by estimating the relative spectral powers (RSPs) across four main frequency bands (delta [0.5 Hz 3 Hz], theta [3 Hz 8 Hz], alpha [8 Hz 12 Hz], and beta [12 Hz 35 Hz]). Across the subjects, the spectral characteristics of the in-ear recordings revealed striking similarities with scalp EEG channels (Figure 6). In particular, the RSPs confirmed a single peak around 10 Hz, approximately aligned across in-ear and scalp channels (Figure 6b for the grand average). In particular, for the different conditions, the power levels in the alpha band remained relatively similar between the in-ear and scalp recordings (no significant differences in power, Figure 6b and Table 3). Also, in both devices, low-frequency activities (delta and theta) prevailed over all other frequency bands.

Nevertheless, the low-frequency power on the scalp remained relatively stable across the three conditions, whereas, for the in-ear recordings, the low-frequency power increased with the introduction of artifacts. Finally, further differences between the scalp and in-ear channels were also identified in the high-frequency beta range (Table 3). This power was significantly higher on the scalp during all conditions compared to the in-ear recordings. Given the broader spectrum of the beta band, we divided it into two frequency domains: beta-1, ranging from 12 Hz to 18 Hz, and beta-2, ranging from 18 Hz to 35 Hz. Regarding beta-1, only C3 showed a significant difference between the in-ear and scalp recordings. For beta-2, all conditions exhibited significant differences between the recording devices.

### 3.4. Sleep Slow Waves and Spindle Correlations

**Figure 7:**
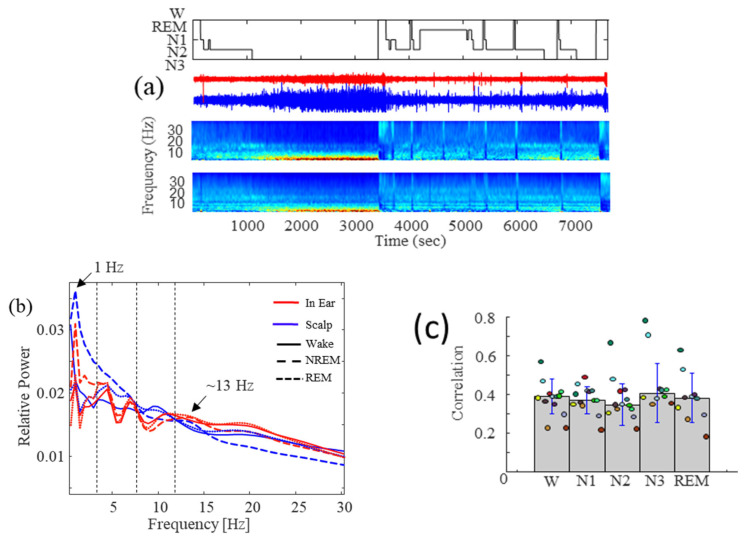
Comparative analysis of the macro-architecture of sleep. (**a**) Hypnogram of a night: raw signals-relative spectrogram of scalp and relative spectrogram of in-ear recordings. (**b**) Spectrogram comparing the relative power of each frequency across different sleep stages. (**c**) Correlation between in-ear and scalp recordings for each stage of sleep (colors of the dots correspond to the colors in Figure 8b).

We conducted full PSGs in eight subjects involving >70 recorded hours of sleep. In all subjects, the spectrograms of the in-ear recordings revealed strong similarities with the scalp EEG channels (Figure 7a). In particular, the relative power spectral densities computed during NREM confirmed a single peak around 1 Hz associated with the presence of strong SW activities, approximately aligned across the in-ear and scalp channels, and the presence of a peak around 13 Hz related to the concomitant occurrence of sleep spindles in the scalp and in-ear recordings (Figure 7b). We confirmed this high similarity through a quantitative analysis carried out on the signals. In particular, as a coarse measure of concordance between the broad-band signals, we computed the mean correlation for each 30 s window and averaged it across sleep stages for each recording session. We found a relatively high correlation (between 0.35 and 0.43) regardless of the sleep phase, indicating that the signals were generally consistent between in-ear and scalp during the whole sleep session (Figure 7c).

**Figure 8:**
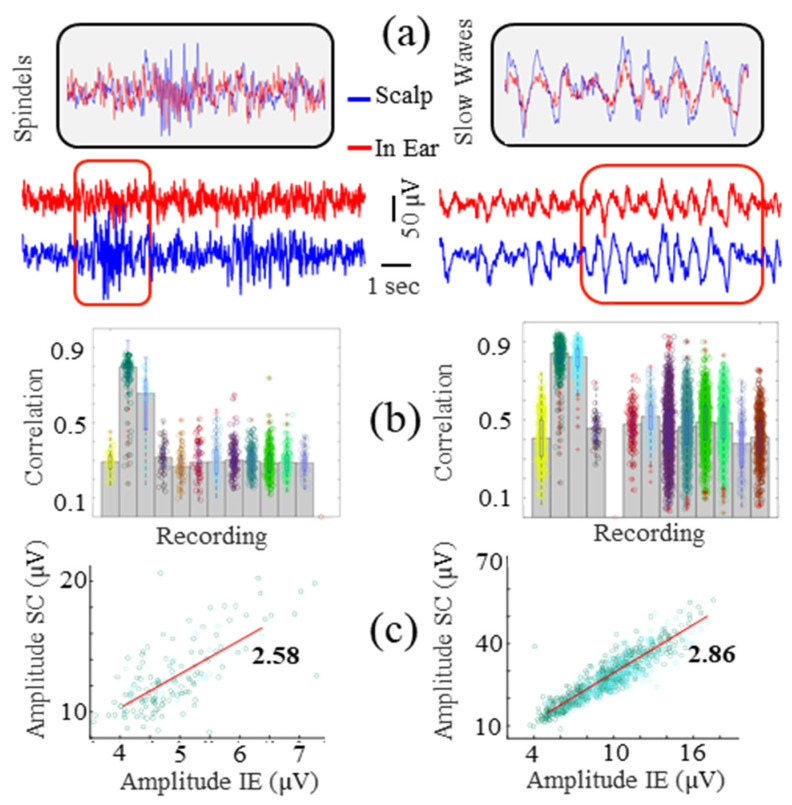
Correlation analysis of spindles (**Left**) and slow waves (**Right**) between the in-ear and scalp EEG signals. (**a**) A 10 s sample of EEG signals during spindles or slow waves at the scalp. (**b**) Pearson’s correlation values for each subject (colors of the dots correspond to the colors in Figure 7c). (**c**) Amplitude plots for scalp (SC) and in-ear (IE) recordings.

To better explore the similarities between individual in-ear and scalp patterns, correlation coefficients were then assessed between the in-ear signal and the scalp EEGs (T7-T8) to quantify the in-ear device’s capability to record sleep SW and spindles. These sleep patterns were identified on the scalp channels during NREM sleep using 6 s windows. When a pattern was detected on the scalp, we computed the linear correlation coefficient with the corresponding synchronized window in the in-ear signal. For SWs, a statistically significant correlation was found in 12 out of 13 recordings (92%), with a correlation of 0.52 ± 0.21 (Figure 8b). Regarding the spindles, the correlations were slightly lower with a correlation of 0.38 ± 0.15, occurring in 12 out of 13 recordings as well (Figure 8b). Notably, two recordings exhibited substantially higher correlations for both SWs and spindles. As in the previous paradigms, we compared the amplitudes of SWs and spindles recorded on both devices, assessing their similarity with the linear regression coefficient. In the two recordings exhibiting the strongest correlations, a linear relationship was identified between SWs and spindle amplitudes recorded on the scalp and in-ear devices, with corresponding factors of 2.86 and 2.58, respectively (Figure 8c). This indicates that, on average, these patterns exhibit amplitudes 2.6 to 2.9 times greater on the scalp compared to the in-ear recordings. These findings are consistent with the results obtained for alpha wave amplitudes.

## 4. Discussion

Currently, scalp EEG systems are the primary tool for studying brain activity in lab conditions. However, with advancements in bioelectronics, several monitoring systems designed for in-ear EEGs have been proposed for real-world deployment and have demonstrated noteworthy results [17,18,19]. Despite this, there is a notable absence of universally agreed-upon benchmarks to define the performance and usability of these new wearable devices. In our study, we present a systematic evaluation of a dry-contact in-ear EEG device developed by Naox Technologies, comparing its effectiveness and reliability against a standard scalp EEG in a group of healthy participants. In particular, our study investigated the capacity of the in-ear device to record several types of physiological and spontaneous EEG patterns. For this, we conducted a comprehensive signal quality analysis, assessing the correlation coefficient between these patterns recorded at temporal scalp locations (bipolar T7-T8) using a standard EEG system and the corresponding in-ear signals. Using this framework, the strong correlations reported in the replayed EEG signals (Section A), alpha waves (Section B), eye artifacts (Section C), power spectra (Section D), sleep slow waves, and spindles (Section E) suggest that the investigated dry-contact ear electrodes successfully captured meaningful EEG signals (see summary Table 4). These findings further imply that the quality of the recordings using the in-ear electrodes is comparable to that of the recordings obtained from conventional wet scalp electrode setups. Overall, throughout our analysis, we found clear similarities in the temporal and spectral characteristics between scalp and in-ear signals. These results are consistent with those of other studies in the literature comparing the similarity between scalp and in-ear signals. In particular, in a study by Looney and co-authors [3], the average correlation between the in-ear electrodes and scalp T7 and T8 was in the range of ~0.7–0.8 for the eyes-closed condition (0.76 in our case). The similarity of the in-ear signal with scalp electrodes at the temporal locations suggests that most of the sources recorded with electrodes in contralateral ear canals originate in the temporal lobe, as has been reported by computational models [44].

Despite this, we observed significant differences between the scalp and in-ear measurements when comparing their respective signal amplitudes. The average scale factor for these deviations ranged from 2.6 to 2.9, indicating that the amplitudes measured at the scalp were consistently 2.6 to 2.9 times higher than those measured by the in-ear electrodes. Similar differences have also been reported by previous studies. For instance, Henao and co-authors [45] reported a reduction in the in-ear amplitude by a factor of ~2.5 compared to a scalp channel. In our case, these differences existed across all recording conditions, strongly suggesting an effect of electrode size and geometry. Moreover, these characteristics of electrode–skin impedance were also considerably influenced by the actual contact surface. Variable contact surfaces occur in different individuals depending on additional parameters, including the geometrical complexity of the ear canal and related contact pressure. Clearly, the lower signal amplitudes observed in the in-ear EEG measurements could impact the accuracy of assessing brain activity, particularly in clinical settings where precise measurements are imperative. Discrepancies in signal amplitudes between the scalp and in-ear EEG recordings may pose challenges in establishing standardized protocols and reference ranges for interpreting EEG data. Clinicians and researchers may need to account for these discrepancies when utilizing in-ear EEG devices for longitudinal monitoring or cross-sectional studies. Further research and validation studies are needed to understand the implications of these disparities comprehensively and to optimize the application of in-ear EEG technology in clinical practice.

Also, hand-inking the in-ear earplugs likely contributed to further electrode size variability. In the future, automated methods like inkjet printing could significantly reduce this variability. Implementing automated manufacturing processes for electrode production can yield numerous advantages, such as enhanced efficiency, scalability, and consistent electrode quality. However, this implementation may also present specific challenges that must be addressed to maintain consistent electrode quality. Variations or inaccuracies in the manufacturing process could potentially result in slight differences in electrode size, shape, or positioning, impacting electrode–skin contact and signal quality. To tackle this challenge, rigorous quality control measures must be enforced throughout the manufacturing process, including regular equipment calibration and meticulous inspection of finished products. Furthermore, selecting suitable materials for electrode fabrication is critical to minimizing contaminants or impurities that could degrade electrode performance. Optimizing manufacturing parameters, such as temperature and pressure, also plays a crucial role in mitigating manufacturing challenges and ensuring the production of high-quality electrodes.

In examining the correlation between the in-ear EEG and scalp EEG measurements, we must note the presence of relatively large inter-subject variability in our findings. A significant factor contributing to this variability could be attributed to the fit of the electrodes within the ear canal, which varies from one subject to another. Moreover, the inherent physiological and anatomical differences among subjects further compound these variations. These differences can influence the efficacy with which the electrodes capture electrical activity, thereby affecting the overall correlation between in-ear and scalp EEG results. Thus, although our findings present a certain trend, the inter-subject variability cautions against a one-size-fits-all interpretation and highlights the need for individualized considerations in applying ear-EEG technology. Recognizing and addressing this variability is crucial to ensure the accuracy and dependability of in-ear EEG measurements across different individuals and populations. The further exploration and refinement of the technology is necessary to tackle these challenges and optimize its effectiveness in a wide range of research and clinical contexts. This may involve the development of customized algorithms or signal processing techniques tailored to individual users, as well as advancements in electrode design and placement strategies. Also, recent research has focused mainly on the materials used for electrodes as well as on the design of the earpieces, with specific emphasis on the shape of the electrodes or sensors with highly flexible or adaptable components [46]. Further design changes to the in-ear system (that facilitate the earpieces to stay in place or enable the sensors to adapt to changes in the shape of the ear) will further improve in-ear recording when used for long-term recordings in an out-of-the-lab setting. Also, by obtaining the precise anatomical data of the participants’ ear canals prior to device deployment, researchers can tailor the design and placement of the electrodes within the ear canal to ensure optimal fit and contact with the skin, thereby minimizing signal artifacts and maximizing signal quality. Nevertheless, previous work has demonstrated that the signal quality from customized earpieces does not significantly surpass that obtained from generic earpieces [47]. Furthermore, researchers may consider incorporating feedback mechanisms into in-ear EEG devices to monitor electrode–skin contact and signal quality during data acquisition continuously. This could involve integrating sensors or impedance monitoring systems into the device design to detect and mitigate issues related to poor electrode contact or signal degradation in real time. In addition to technological considerations, researchers may also implement standardized protocols for electrode placement and signal acquisition to ensure consistency and reproducibility across participants. This could involve providing training and guidelines for device fitting and positioning to research participants or healthcare professionals responsible for device deployment.

Furthermore, our findings reveal distinct effects of each condition, inducing artifacts on scalp and in-ear signals. Generally, we predominantly observed significant deteriorations in in-ear signals during face/head movements within the low-frequency (delta/theta) bands. Consistent with prior research [23], we hypothesize that these pronounced artifacts of in-ear signals were likely motion artifacts stemming from alterations in the shape of the ear canal due to jaw and head movements. Addressing these challenges may necessitate the development of robust signal processing algorithms [48] or hardware modifications to minimize the impact of motion artifacts and enhance the overall performance of in-ear EEG devices in dynamic environments [49]. It is also conceivable that employing highly stretchable, soft silicone elastomers in constructing the earpieces could potentially mitigate some of these motion artifacts, enabling the earpiece to adapt to changes in the shape of the ear canal. Furthermore, myogenic artifacts related to jaw muscle contractions were present all over the scalp and in the ear, with the highest deterioration in the high-frequency beta band. However, these deviations were generally more pronounced at the scalp compared to the in-ear signals. We speculate that this discrepancy may be linked to tension in the jaw muscles, particularly notable in the lateral temporal regions due to the proximity of the temporalis muscles. Nonetheless, our evaluation has shown that high-quality in-ear EEG signals can be reliably captured during specific periods, particularly during resting or quiet moments, such as sleep. These periods offer a unique opportunity to observe significant neural events in a stable and controlled environment. Despite the challenges posed by motion artifacts, harnessing these critical periods, such as during rest or sleep, can still provide valuable insights into brain activity using in-ear EEG technology.

Our paper details a thorough examination that includes emulated signal tests and comparisons of EEG brain waves. The high correlation levels observed in most subjects suggest that the in-ear system is as effective at detecting spontaneous EEG oscillations as the traditional scalp wet electrode systems. Future research may expand on a variety of other stimuli-evoked paradigms, including visually evoked potentials, mismatch negativity (MMN), P300, and, more importantly, the auditory steady-state response (ASSR), which is often used to assess novel ear-EEG devices [5,6]. By comparing the amplitude, latency, and waveform features of these potentials recorded via in-ear EEG against those captured by a traditional scalp EEG, we can gain insights into the device’s efficacy in capturing neural components associated with sensory processing, attention, and cognitive decision-making processes. In addition to adopting various other paradigms, the population size can also be expanded to younger or older participants.

## 5. Conclusions

Our paper provides a comprehensive analysis focused on evaluating the signal quality of a specific wearable in-ear EEG device. It systematically assesses its performance relative to conventional scalp EEG systems. Our evaluations demonstrate that the ear device can reliably capture EEG signals across various physiological conditions and activities, including alpha waves, eye movements, and sleep patterns, with correlations to scalp EEG signals ranging from moderate to almost perfect.

Moreover, we found notable deviations between scalp and in-ear amplitudes and the impact of artifacts on signal quality. These results underscore the importance of continued refinement in electrode design and deployment. The variability in electrode–skin contact, influenced by individual anatomical and physiological differences, suggests a need for further research to optimize electrode fit and signal acquisition in a broader demographic.

Future studies should expand the evaluation framework to include a wider range of EEG patterns, both spontaneous and stimulus-evoked. Implementing automated manufacturing processes for electrode production could also reduce variability and improve the reliability of wearable EEG systems. By addressing these considerations, the field can advance toward producing wearable EEG technologies that offer both high-fidelity brain monitoring and practical usability outside of clinical settings.

## Figures and Tables

**Table 1:** Technical parameter summary of the NAOX in-ear device.

Features	NAOX Earbuds
Skin Contact Location	Ear canal
Electrodes	2 actives and dry silver electrodes by ear tip
Channel	1 single bipolar EEG channel left ear–right ear
Sampling Frequency	250 Hz
Transfer of Data	Bluetooth 2.4 GHz
Data Format	The European Data Format (EDF)
Autonomy	~10 h
Weight	20 g
Input noise	<6 μV peak to valley
CMRR	>80 dB at 50 Hz

**Table 2:** Experimental protocol.

Tasks	Duration ^1^	Condition
Alpha paradigm	5	C1
Artifact paradigm	2	C3
Hyperventilation	2-3-2	C3
PVT	10	C2
Reading text	10	C3
Emotion recognition	5	C2
Relaxation period	10	C1

^1^ Durations are noted in minutes.

**Table 3:** Statistical analysis.

IE vs. Scalp *p*-Value	Delta	Theta	Alpha	Beta-1 (12–18 Hz)	Beta-2 (18–35 Hz)
C1	0.00625	0.029375	0.648125	0.548125	0.001875
C2	0.006875	0.00062461	0.20375	0.775625	0.00062461
C3	0.0025	0.00062461	0.935	0.039375	0.00062461

**Table 4:** Summary table of the correlations between in-ear and scalp EEGs.

Pattern	Number of Recording	Correlation Mean ± Std	Statistically Significant Cases (*p* < 0.05)
Seizures	45	0.87 ± 0.08	45/45
Non-Seizures	45	0.86 ± 0.09	45/45
Alpha waves	18	0.76 ± 0.19	15/18
Artifacts	18	0.87 ± 0.19	18/18
Spindles	12	0.38 ± 0.15	11/12
Slow waves	12	0.52 ± 0.21	11/12
Sleep phases	8	0.39 ± 0.11	8/8

## Data Availability

The EEG datasets generated for this study are available upon request from the corresponding author.
